# 1017. Case Study. Venovenous ECMO in Toxic Epidermal Necrolysis-related Respiratory Failure

**DOI:** 10.1093/jbcr/irag033.285

**Published:** 2026-04-06

**Authors:** Jan V Stevens, Jude Jaraki, Yusuke Terasaki, Heather Dolman, Michael T White, Andrew Isaacson

**Affiliations:** Wayne State University School of Medicine, Grosse Pointe Park, MI; Wayne State University School of Medicine, Detroit, MI; Wayne State University School of Medicine, Detroit, MI; Detroit Receiving Hospital Burn Center, Detroit, MI; Detroit Receiving Hospital Burn Center, Detroit, MI; Wayne State University School of Medicine, Detroit, MI

## Abstract

**Patient Presentation (age range, injury details, relevant history):**

A 33-year-old woman was transferred to our ABA-verified burn center with a 5-day history of a new onset blistering rash over the trunk and bilateral arms. She had recently taken both ibuprofen and acyclovir due to concern of an HSV-2 flare-up. Physical exam showed additional desquamating lesions of the head, neck, and bilateral legs along with genital, ocular, and oral mucosal involvement. Her estimated initial body surface area (BSA) affected was 30%.

**Clinical Challenges:**

The severity and distribution of the rash led to a clinical diagnosis of toxic epidermal necrolysis (TEN). Although initially presenting as hemodynamically normal and afebrile, the patient complained of worsening dyspnea on admit day 2. Her supplemental oxygen demands increased despite dexamethasone treatment for suspected airway edema and she was intubated. By admit day 4, her chest x-ray showed worsening bilateral patchy opacities with concern of acute respiratory distress syndrome (ARDS). Concomitantly, her BSA affected rapidly progressed to 85% by this time despite local wound care.

**Management Approach:**

The patient underwent 4 days of IV immunoglobulin, 7 days of cyclosporine, and 4 plasmapheresis treatments with marked improvement of her epidermal disease. However, intermittent desaturations and hypoxia persisted. Positive end expiratory pressure was increased from 5 to 12 cm H2O and we initiated neuromuscular blockade to assist with ventilator synchrony. Her hypercarbia worsened with paCO2 ranging from 50-70 mmHg and peak inspiratory pressures increased to 35-45 cm H2O. Her open wounds and wound care needs precluded proning, but her BSA affected decreased to an estimated 4%. She was cannulated for venovenous extracorporeal membrane oxygenation (VV-ECMO) on admission day 13 following a failure to respond to maximal ventilator settings.

**Outcomes:**

A tracheostomy was performed on admit day 16/VV-ECMO day 4. Her clinical status improved and she was decannulated from VV-ECMO on admit day 21/VV-ECMO day 9. The patient’s ventilator requirements were weaned until she was ventilator-independent by hospital day 32 with 31 total ventilator days. Her wounds were completely re-epithelialized by hospital day 55 and total length of stay was 113 days due to complications from malnutrition, multifactorial liver failure, and Stenotrophomonas pneumonia.

**Lessons Learned:**

TEN presents as a severe cutaneous and/or mucosal adverse reaction with >30% BSA affected; however, this disease can result in pulmonary complications such as bronchial sloughing, pneumonitis, airway edema, and subsequent ARDS. VV-ECMO presents a unique oxygenation modality for patients with respiratory sequelae of TEN unresponsive to mechanical ventilation, paralysis, or steroids.

**Applicability to Practice:**

Though rare, pulmonary complications of TEN can present even as a patient's epidermal disease resolves. These cases can progress to life-threatening ARDS. When traditional therapies fail, providers should consider VV-ECMO as an adjunct for pulmonary support for these patients.

